# Dendritic cells: the yin and yang in disease progression

**DOI:** 10.3389/fimmu.2023.1321051

**Published:** 2024-01-04

**Authors:** Carlos Jiménez-Cortegana, Francisca Palomares, Gonzalo Alba, Consuelo Santa-María, Luis de la Cruz-Merino, Victor Sánchez-Margalet, Soledad López-Enríquez

**Affiliations:** ^1^ Department of Medical Biochemistry, Molecular Biology and Immunology, School of Medicine, University of Seville, Seville, Spain; ^2^ Department of Biochemistry and Molecular Biology, School of Pharmacy, University of Seville, Seville, Spain; ^3^ Clinical Oncology Dept. Medicine Department, University of Seville, Virgen Macarena University Hospital, Seville, Spain

**Keywords:** dendritic cell, inflammation, immunity, cancer, inflammatory bowel disease

## Abstract

Dendritic cells (DCs) are antigen presenting cells that link innate and adaptive immunity. DCs have been historically considered as the most effective and potent cell population to capture, process and present antigens to activate naïve T cells and originate favorable immune responses in many diseases, such as cancer. However, in the last decades, it has been observed that DCs not only promote beneficial responses, but also drive the initiation and progression of some pathologies, including inflammatory bowel disease (IBD). In line with those notions, different therapeutic approaches have been tested to enhance or impair the concentration and role of the different DC subsets. The blockade of inhibitory pathways to promote DCs or DC-based vaccines have been successfully assessed in cancer, whereas the targeting of DCs to inhibit their functionality has proved to be favorable in IBD. In this review, we (a) described the general role of DCs, (b) explained the DC subsets and their role in immunogenicity, (c) analyzed the role of DCs in cancer and therapeutic approaches to promote immunogenic DCs and (d) analyzed the role of DCs in IBD and therapeutic approaches to reduced DC-induced inflammation. Therefore, we aimed to highlight the “yin-yang” role of DCs to improve the understand of this type of cells in disease progression.

## 1 Introduction

**Figure 1:**
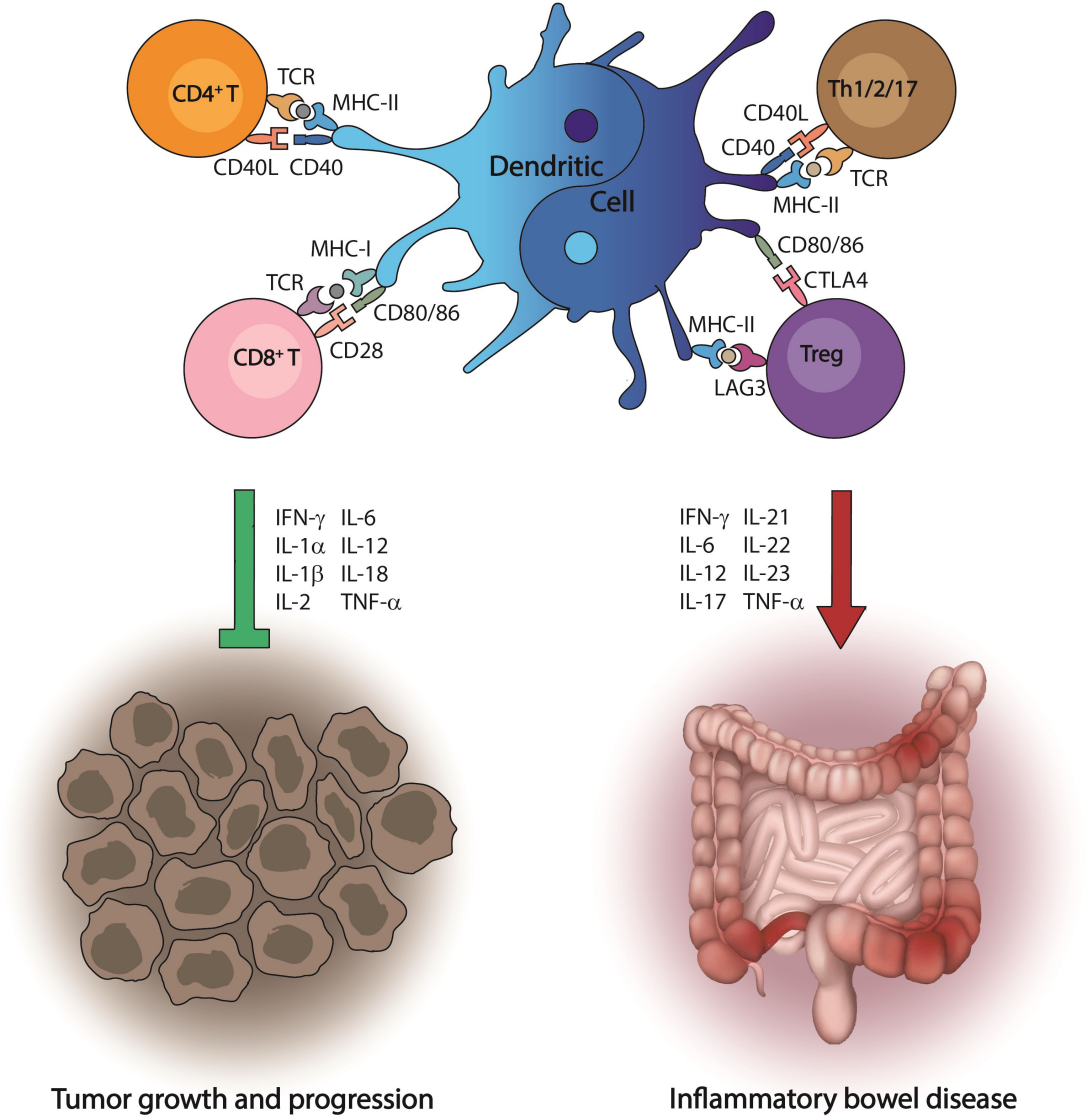
Dual role of dendritic cells in disease progression. Dendritic cells are the most effective antigen-presenting cells to initiate immune responses, which lead to the activation of the cytotoxic machinery driven by T cells. This event is crucial to recognize tumor antigens and kill cancer cells. However, in the environment produced by gut inflammation, dendritic cells have an opposite behavior and promote a cascade of proinflammatory signals that ultimately induce inflammatory bowel disease.

Dendritic cells (DCs), the bridge between innate and adaptive immune responses, are considered as the most potent antigen presenting cells (APCs) since they control both T cell immunity and tolerance (1). DCs represent a heterogeneous cell population which is differentiated from CD34^+^ hematopoietic precursors into other developed DC precursors. DCs comprise subsets in both lymphoid and nonlymphoid tissues, such as monocyte-derived DCs (moDCs) or inflammatory DC (infDC), plasmacytoid DCs (pDCs), and conventional DCs (cDC) 1 and cDC2s (also known as myeloid or classical DCs) (2). DCs mainly induce immune responses by capturing, processing and presenting unknown or self-antigens to adaptive immune cells. External antigens derive from diseases such as viral infections (3),or cancer (4), and self-antigens take part in autoimmune diseases (5), which comprise a set of disorders including (but not limited to) allergies, brain diseases, or inflammatory bowel disease (IBD) (6–8). However, in the last decades, it has been described that DCs have an interesting opposite behavior depending on their environment. That means, DCs have found to be widely downregulated in many diseases such as cancer where are called tolerogenic DCs (9), playing an important role in inducing peripheral tolerance via specific mechanisms, as activation of Treg cells (10), suppression of effector T cells, and negative modulation of Th1/Th2 immune responses. For this, DC has been successfully targeted by using inhibitory drugs or DC-based vaccines as immunotherapies (11, 12), whereas DC activation can play a contributing role in the pathogenesis of other disorders, including IBD (13) (see **Figure 1**). Of note, DCs have also demonstrated both beneficial and detrimental functions within the same disease (14).

The reasons why DCs have a ‘yin and yang’, dual role are not still completely elucidated and may have important implications in terms of therapeutic approaches. In this regard, it is known that the transcription factor β-catenin induces different characteristics over human moDCs in a dose-dependent manner, since its inhibition leads to a pro-inflammatory state by increasing IL-12p70 and reducing IL-10, whereas its activation enhances the nuclear β-catenin, which is associated with low levels of IL-12p70, higher IL-10 and the expression of inhibitory cell markers on DCs (15). C-type lectin receptors (CLRs) can also drive different behaviors in DCs. In fact, different nanovaccines have been designed to act on the CLRs from DCs and induce a regulatory response in allergic patients (16). Specifically, DC-specific ICAM-3–grabbing nonintegrin (DC-SIGN, also known as CD209) is a CLR that binds the nanovaccines changing the DC phenotype and improving Treg proliferation (17).

In turn, DC-SIGN also promotes the serine/threonine protein kinase RAF1 that may participate in the activation and proliferation of T cells since the inhibition of RAF kinases could impair DC activation in both human and mice, thus compromising T cell-mediated immune responses (18). Then, we aimed to review the dual behavior of DCs in two different microenvironments, cancer and IBD, putting the focus on their dysregulation and their pro-inflammatory function, respectively.

## 2 DC subsets and their role in immunogenicity

### 2.1 Immunogenic and tolerogenic role of DCs

Immunogenicity is the ability of proteinic substances (e.g., foreign antigens) to promote immune responses. Currently, it is known that DCs activate their immunogenic machinery *ex profeso* to sample antigens by phagocytosis, receptor-mediated endocytosis, or micropinocytosis, and processing them for presentation to CD4^+^ T cells on major histocompatibility complex class II (MHC-II) molecules and CD8^+^ T cells on MHC-I (19). However, the discovery of the immunogenic capacity of DCs has been shown to be suitable for cancer therapy (20) and for other pathologies (21). In the last years, these immunogenic DCs have been used in personalized treatments in patients with HIV receiving antiretroviral treatment (22) or in ovarian cancer patients (23).

Another relevant aspect regarding immunogenicity is DC maturation, since mature DCs use different molecular mechanisms to tailor immune responses depending on the stimulus (24). In this sense, mature DCs require a set of receptors to have immunogenic properties including (but not limited to) CD31, CD40, CD80, CD83 and CD86 (25).

In addition, DCs have a tolerogenic ability in different immunological diseases (1). In this sense, DCs take part in the central and peripheral tolerance by controlling effector and regulatory mechanisms, especially self-reactivity associated to autoimmunity (26). However, another tolerogenic effect of DCs is their capacity to differentiate T cells into their regulatory phenotype (Tregs) (27). Those functions are carried out by immature or semi-mature DCs characterized by the expression of surface markers such as PD-L1 or CTLA-4 and the downregulation of MHC molecules, CD40, CD80 or CD86 (28, 29). Also, it has been found that the production of anti-inflammatory cytokines (e.g., IL-10 and TGF-β) by DCs induces tolerogenic effects in this cell population (30).

### 2.2 DC subtypes

Different DC subsets have been found depending on their location in different tissues and lymphoid organs, including lymph nodes, spleen, thymus, gut, blood, or skin (31). In this regard, DC plasticity makes their categorization difficult, but a simplified classification based on the ontogeny divides this cell population into cDCs, moDCs or infDCs, pDCs, and Langerhans cells (LCs) (19), which could have immunogenic or tolerogenic effects.

In steady state, cDCs are in both non-lymphoid tissues and the spleen marginal zone and have a high capacity to migrate to T-lymphocyte zones (TLZs) of lymph nodes also during inflammation (32). cDC1s can be found within the lymph node paracortex and uptake cell-associated antigens (also dead cells) via receptors, such as DEC205 (also known as CD205) or T cell immunoglobulin and mucin domain-containing protein 3 (TIM3), preferentially by cross-presentation on MHC-I to CD8^+^ T cells, an essential pathway for both antiviral and antitumor immunity (33). cDC1s are also characterized by high expressions of CD103 and toll-like receptor (TLR) 3 (34), contribute to intracellular protection of T helper (Th) 1 cells by producing interleukin (IL)-12 (35), and promote Th17 responses in against influenza virus infection (36). cDC2s uptake antigens in the skin and migrate to TLZs by different pathways, including C-X-C Motif Chemokine Receptor 4 (CXCR4)- or CC-chemokine receptor 7 (CCR7)-dependent manners. In addition, cDC2s also uptake and cross-present tumor-associated antigens (TAAs) under certain conditions (33), and also express interferon regulatory factor 4 (IRF4), which makes cDC2s particularly efficient for antigen processing and presentation on MHC-II, thus inducing superior CD4^+^ T cell proliferation compared with cDC1s (33, 37) and supporting Th2 and Th17 polarization (38). Interestingly, the colony stimulating factor-1 (CSF-1), which is found in the airway and alveolar microenvironment, upregulates the expression of CCR7 on cDC2 (but not cDC1) in a IRF4-dependent manner in response to allergen stimuli, promoting Th2 responses (39). In turn, the deletion of IRF4 has been found both to promote and inhibit Th17 responses (40).

Similar to IRF4 in cDCs, moDCs have demonstrated dual roles by activating anti-tumoral CD8^+^ T cells (41) and suppressing respiratory CD8^+^ T cell memory in viral immunity (42). Moreover, moDCs or infDCs and cDC2s express CD11b during inflammation, making these cell populations phenotypically difficult to distinguish (19). Human pDCs are based on the coexpression of CD123 and CD303, whereas mouse pDCs are B220^+^ and CD11c^+^. pDCs have specific functions because they can recognize RNA and DNA viruses through TLR-7 and -9, leading to cell activation, and release high amounts of type I interferon (IFN-I) (43). In addition, pDCs not only have demonstrated to play a key role in the development of acute colitis and development of IBD, showing differences in the distribution, phenotype and function in patients with Crohn’s disease (CD) and ulcerative colitis (UC) (13), but also take part in viral infections together with cDC1s (44) by inducing IFN-I and recruiting natural killer (NK) lymphocytes (45). Additionally, LCs are in the epidermis but share common ontogeny with macrophages. LCs are needed for specific adaptive immune responses when antigens are highly found in the epidermis (46), and selectively promote the expansion and activation of skin-resident regulatory T cells (Tregs) to maintain the skin immune homeostasis (46, 47).

## 3 DCs and cancer

Cancer is one of the most common causes of death worldwide (48). Although the cancer death rate is continually falling every year, it was expected to have almost 2,000,000 new cancer cases and more than 600,000 cancer deaths in United States during the last year (49). As explained above, DCs acts as professional APCs to initiate immune responses. In cancer, the immunogenic capacity of DCs is called “cancer-immunity cycle”, a multistep and metabolic mechanism that explains how DCs capture, process and present TAAs to naïve T cells, which are consequently activated and infiltrated within the tumor microenvironment (TME) to kill cancer cells by specifically recognizing similar antigens, leading to the release of new TAAs and making the process starts over (50). However, inhibitory factors affect anti-tumor DC activity, including (but not limited to) tumor growth factor (TGF)-β (51), IL-10 (52), cytotoxic T-lymphocyte antigen (CTLA)-4 (53) and programmed cell death protein (PD)-1 expressions (51, 53), thus leading to cancer progression.

### 3.1 ‘Yin-yang’ role of DCs in cancer

Of all existing types of DCs, pDCs and moDCs seem to have contradictory roles in cancer immunity. Evidence has demonstrated that pDCs infiltrate different types of tumors and are associated with poor outcomes (54), due to the expression of inhibitory markers including lymphocyte-activation gene (LAG)-3, PD-1, and CTLA-4 (55–57), the release of immunosuppressive cytokines (e.g., IL-10, TGF-β, and prostaglandin E2) (58) as well as the expansion and Treg accumulation (59). However, it has been suggested that pDCs may have a lytic ability against tumor cells (60, 61). MoDCs are phenotypically similar to antitumoral cDCs (62), and have demonstrated to mediate beneficial immune responses (63, 64), but are also involved in the maintenance of Th17 responses, which could induce a pro-tumoral state (65), and are related to monocytic myeloid-derived suppressor cells (MDSCs) (66), which have been correlated with tumor progression and poor outcomes in many oncological settings (67–69).

By contrast, cDCs have demonstrated a preferential capacity to promote antigen presentation to T cells in cancer (70). Specifically, cDC1s, which are characterized by the expression of integrin aE (also known as CD103) in mice or BDCA3 (CD141) in humans (31), have a superior ability to transport TAAs to the draining lymph node and cross-present antigens on MHC-I to activate cytotoxic T cells (71). The key role of cDC1s in cancer have been extensively supported by many studies in both humans and murine models. For example, the presence of cDC1s has been correlated with good outcomes in melanoma patients using anti-PD-1 (72). Mice lacking CD103^+^ cDC1s have driven an impaired CD40L-overexpressing chimeric antigen receptor (CAR) T cell antitumor response (73). Also, Batf3 DCs have shown to be necessary for effective antitumor responses driven by monoclonal antibodies and adoptive T cell therapy (74). On the contrary, cDC2s have a reduced capacity to cross-present antigens to CD8 T cells and are more efficient by priming CD4 T cells to induce antitumor immunity (37). Migratory CD301b+ cDC2s have demonstrated to be essential for an effective CD4 T cell priming (75). However, despite these well-established notions, it has been recently known that the deletion of MHC-II and CD40 in cDC1 also prevented early CD4 T cell priming and impaired tumor rejection in fibrosarcoma-bearing mice, thus suggesting that cDC1s are also required for CD4 T cell priming against TAAs (76).

## 4 Therapeutic approaches to promote immunogenic DCs in cancer

### 4.1 Cancer immunotherapies

Immunotherapies have demonstrated to improve outcomes in many different types of cancers (77, 78). Specifically, the infiltration of DCs into tumors has been positively correlated with prognosis and survival (79, 80), which has made the design of different therapeutic approaches possible to increase both concentration and functionality of DCs.

Inhibitory pathways and signals have been targeted since they maintain low concentrations of DCs within the TME and lead to tumor progression. Those mechanisms can be inhibited due to the immunosuppressive conditions found in the TME, MDSCs have the ability to decrease antitumor immunity (81). However, the PD-1/PD-L1 immune checkpoint also impairs the activation, proliferation, and cytotoxic function of T cells (82), which has been successfully reverted by using anti-PD-1 therapies, especially when combined with other treatments (83, 84). In addition, it has been observed that DCs could be necessary to boost anti-PD-1 efficacy due to the production of IFN-γ and IL-12 by this cell population (85). Another inhibitory signal, vascular endothelial growth factor (VEGF), has potent antiangiogenic properties and blocks DC maturation and proliferation (86, 87). Therefore, the inhibition of VEGF with targeted anti-VEGF therapies not only prevents angiogenesis, but also improves the capacity of DCs to carry out effective anti-tumor responses (88). IL-6 is another cytokine that promotes cancer progression by up-regulating different pathways that involve apoptosis, angiogenesis, invasiveness, metastasis, or tumor cell metabolism, among others. In fact, it has been reported that IL-6 inhibits anticancer immune responses generated by cytotoxic chemotherapy (89), and promotes breast cancer metastasis suppressing the anti-tumor immune response via IL-6/JAK/STAT3 signaling (90). In line with this notion, IL-6/JAK/STAT3 signaling has demonstrated to be a promising therapeutic target for hepatocellular carcinoma (91). On the contrary, although it has been shown that IL-10 levels are altered in cancer as well as IL-4 and IL-35 (92), there is evidence that IL-10 has dual functions in cancer (93). In this line, IL-10 suppression enhances T cell antitumor immunity and responses to checkpoint blockade in chronic lymphocytic leukemia (94).

### 4.2 Cancer DC-based vaccines

Other alternatives to improve anti-tumor immunity are DC vaccines, that have been clinically evaluated and considered as safe therapies due to limited toxicities either alone or combined with other treatments (95–98). DCs are considered as the most effective APCs and promote immunological T cell response (33). Altogether, those characteristics made DCs as the most appropriate cell population for the development of cancer vaccines. Specifically, cDC1 vaccines have shown better anti-tumor efficacy compared with MoDC vaccines (99, 100), whereas another study reviewed that not only cDC but also pDC vaccines may be considered as more potent alternatives compared with MoDC vaccines (101). Another promising immunotherapeutic approach is the so-called *in vivo* vaccination, which consist of targeting DCs with DC receptor ligands, adjuvants, or other types of molecules that can accurately bind to DCs to exert better effects on anti-tumor T cell responses (102). *In vivo* vaccines target DC receptors such as TLRs (103), or adenosine receptors (104), and has concluded with promising results (105). Moreover, it has been demonstrated that DCs-pulsed with sulforaphane, a natural compound presents in cruciferous as broccoli induce T-cell activation through the modulation of regulatory molecules, JAK/STAT3- and microRNA-signaling in healthy conditions and in context of pancreatic cancer-derived antigens, proposing the possibility to use the sulforaphane in the co-treatment of cancer (106).

## 5 DCs and inflammatory bowel disease

IBD is a disorder with high incidence (around 3.5 million people in the last decade only considering North America and Europe) and prevalence (currently exceeding 0.3%) (107). The etiology of IBD remains unclear, although it is known that involves the interaction between immune responses with genetic, environmental, and microbial factors, geographical location, or an inappropriate diet (108, 109). IBD is characterized by an altered epithelial barrier function due to exacerbated and continuing immune reactions toward the microbiota, including an improved chronic relapsing, and the inhibition of both adequate containments of luminal microorganisms and the ability to absorb nutrients (110). Specifically, UC involves aspect of the colon starting with mucosal inflammation in the rectum. Its main symptoms are bloody diarrhea, abdominal pain, fecal urgency, and tenesmus (111), whereas CD involves the whole gastrointestinal tract (although distal ileum is the most frequently affected part). CD presents periods of flares and remissions and causes transmural pleomorphic inflammation, leading to fistulas, abscesses, and granulomas (112).

### 5.1 Inflammation-associated factors in IBD

Traditionally, it has been believed that gut inflammation has been only promoted by T helper cells (Th) 1, Th2, Th17 and Tregs, but now we known that inflammation is also induced by other immune cells, cytokines and processes, including macrophages, DCs, tumor necrosis factor (TNF), inflammasome activation, or autophagy (113–117). Particularly, autophagy deficiency decreases antigen sampling, increases DC maturation, and promotes pro-inflammatory DCs (118). Atg16l1 autophagy gene deficiency promotes the bacterial translocation of DSS-induced colitis *in vivo* and regulates autophagy and phagocytosis, which lead to an exacerbation of the intestinal inflammation (119). The immune microenvironment of UC inflamed colon is composed not only by follicular Th cells and IL17A^+^ Tregs, but also by memory cells (CD4^+^ T, IL17A^+^CD161^+^ T, and B cells), HLA-DR^+^CD56^+^ granulocytes, M1 macrophages, activated mast cells, neutrophils, and both resting and activated DCs (120, 121). In CD patients, peripheral blood mononuclear cells have high expression of IL-1B in the Treg, DC and monocyte fractions (121), whereas the inflamed mucosa of those patients is characterized by IL-1B in HLA^-^DR^+^CD38^+^ T cells, TNF^+^IFN-γ^+^ naïve B cells, and pDCs (121).

#### 5.1.1 DCs as an inflammation-associated factor in IBD

**Figure 2:**
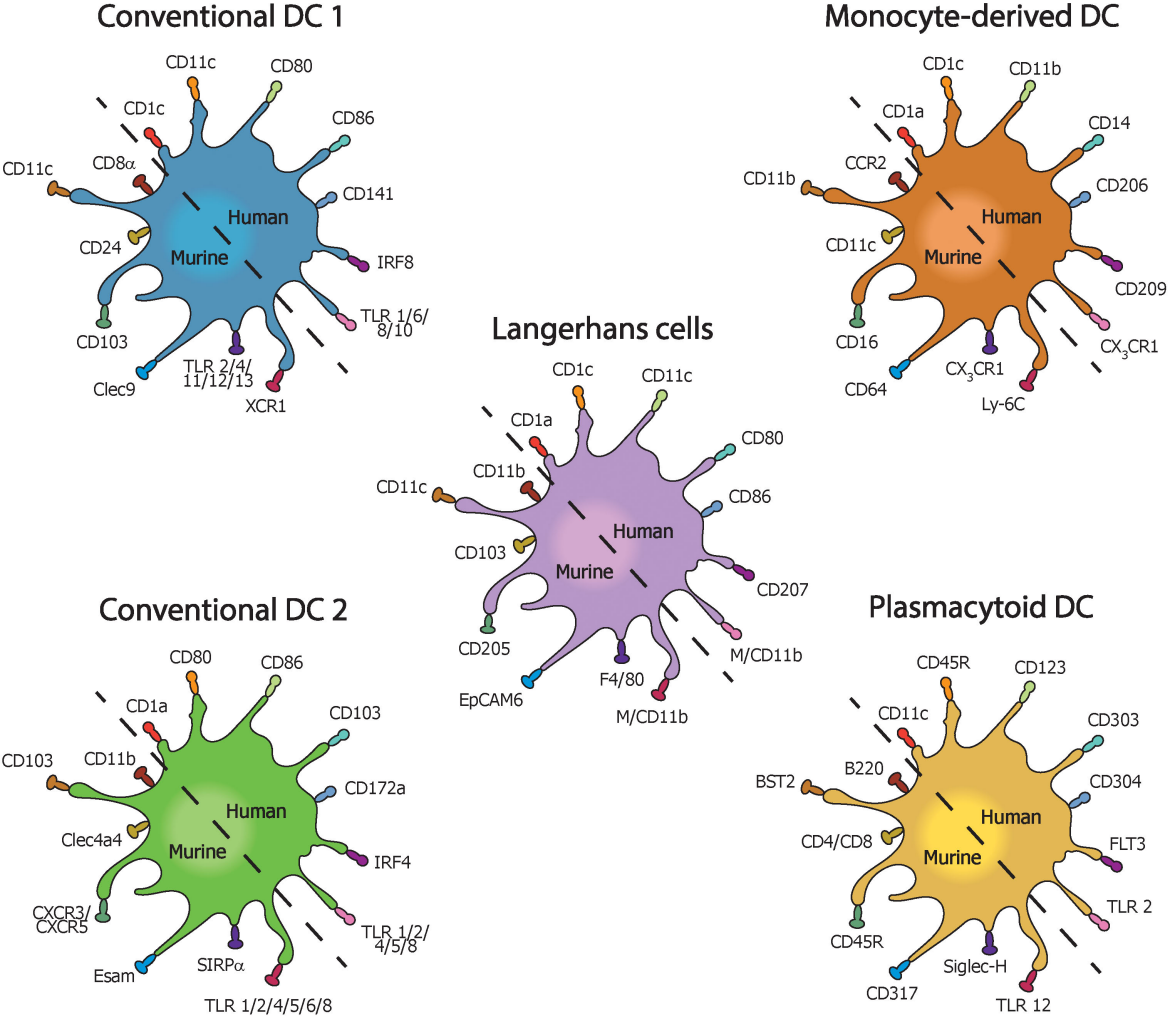
Types of dendritic cells with their most common pro-inflammatory surface markers in mice and humans with inflammatory bowel disease.

DCs promote IBD by expressing different markers in both human and mice (**Figure 2**). cDC subsets have been observed in gut mucosa from both human and mice in steady state (122), although the interaction with T cells to initiate immune responses seems conflicting (123), which could imply that some cell populations and cytokines play a dual role in the pathogenesis of IBD. In line with this notion, TGF-β not only has shown to play an unfavorable function by increasing collagen production and regulating fibrosis in CD patients with stricture (124) but also has been suggested to be necessary to inhibit inflammation in IBD (125). The role of TNF-α is essential in the immunological response of IBD (126). In fact, TNF-α regulates IL-22BP expression by colonic DCs and dampened IL-22-driven restitution of colonic epithelial functions in model of experimental colitis (127). Of note, DCs stimulated *in vitro* with TNF-α could not improve the activation and maturation of DCs compared with *E. coli*-stimulated DCs, which may suggest altered interactions between DCs and intestinal microflora in patients with UC and CD (116).

Intestinal SIRPα^−^CD103^+^ cDC1 constitute a small cell population in the human intestine and are molecularly similar to cross-presenting CD141^+^CLEC9A^+^CADM1^+^ cDC1, whereas SIRPα^+^CD103^+^ cDC2 are the main population in the small intestine in both mice and humans, and SIRPα^+^CD103^−^ cDC2 predominate in the colon. CD103-expressing DCs have also been found in CD patients (128). CD103^+^ DC subsets with high expression of PD-L1/2 could be induced by the deficiency of the Smad7 protein, a negative regulator of TGF-β signaling (129). Also, CD103^+^ DCs have a colitogenic role and show impaired ability to produce Tregs (130) but generate Th1/Th2/Th17 responses through the induction of a huge variety of cytokines, including IFN-γ, IL-13, IL-6, IL-23, IL-12p35, IL-12p40, and TNF (131–133). The CD83 expression in DCs have also been found in IBD patients (134). Moreover, high expression of Receptor-interacting serine/threonine kinase 2 (RIPK2) signaling in DCs is a new therapeutic target in IBD (135, 136). In turn, the upregulation of other molecules, such as CXCL10 and CCL3 were positively correlated with activated DCs in CD and UC (128, 137), postulating them as effective diagnostic biomarkers in IBD. These molecules were found in UC patients compared with their CD counterparts following the exposure to cigarette smoke extract (CSE) *in vitro*, thus demonstrating that CSE modulates DC phenotypes and alters DC function, which results in Th1 polarization and increased levels of Foxp3^+^CD4^+^ T cells in UC (138). Of note, mutations (e.g., in *NOD2fs* gene) also promote a dysfunctional phenotype in DCs and increase CD susceptibility (139).

CD14^+^ cDCs have been found in the inflamed mucosa of IBD patients but are limited in mesenteric lymph nodes (MLNs), which have been less studied in relation to the DC phenotypes (122). By contrast, during the early stage of murine IBD, high levels of both mDCs and pDCs expressing CD11b and B220 have been found in colon (approximately, 50% of each subset) and MLNs (mainly mDCs) (140), but high levels of pDCs in MLNs has also been reported in other cases (141). Conversely, CD14^-^HLA-DR^int^ pDCs have shown to be the most abundant subset in MLNs either expressing CD11c^+^ or not (122), especially in CD (141). High expression of CD11c have also been observed in CD103^+^ DCs (13) and CD123^–^HLA-DR^+^ DCs, which produce more IL-23 in CD patients than in their UC counterparts (142), demonstrating the importance of CD11c in the pathogenesis of IBD. In this sense, the chromofungin (CHR), a chromogranin-A derived peptide, CHR has demonstrated protective properties against intestinal inflammation by regulating CD11c of DCs (143). More CD14^-^ DC subsets have been found in IBD by using transcriptomic analysis and high-dimensional phenotypic mapping, such as CD14^−^CD64^dim^CD11b^+^CD36^+^CD11c^+^ and CD14**^−^** CD64**^−^** CD163**^−^** DCs (141).

In addition, both CD103^+^ and CD11c^-^ DCs have been associated with different levels of TLR expression (144). UC-derived DCs secrete pro-inflammatory cytokines and chemokines via activation of TLRs. TLRs induce infiltration of polymorphic neutrophils and activation of other innate immune cells, as well as the differentiation of naïve T cells to Th1 cells and the activation of DC to release IL-12 (140). In connection with this, regulatory or tolerogenic DCs increase the levels of colon-infiltrated Tregs and inhibited Th1 and Th17 cell-driven colon inflammation in a Galectin-3:TLR-4:Kynurenine-dependent manner, which demonstrated the importance of both TLR and galectin 3 in the immunosuppressive functions of Tregs (145). Similarly, another protein of the galectin family, galectin 1, has demonstrated to limit the immunogenic activity of DCs in a murine IBD, such as CD and UC (146).

## 6 Therapeutic approaches to inhibit DCs in IBD

DCs are emerging as central players since its levels tend to increase in the colonic mucosa and could have important functions in regulating response to gut microflora (13). There is a wide variety of cell-based therapies to improve outcomes in IBD (147), but we will focus on treatments that modulate or reduce DCs producing tolerogenic DCs (**Table 1**).

**Table 1:** Effects on DCs caused by different therapeutic approaches in inflammatory bowel disease.

Type of therapy	Name	Effects on DCs from IBD	References
Antibodies	Vedolizumab	cDC and pDC localization was blocked in the intestinal epithelium	(148)
	Risankizumab	IL-12/IL-23 p40, which is produced by DC, was blocked	(133)
	Ustekinumab	IL-23 p19, which is produced by DCs, was blocked	(133)
	Natalizumab	pDC level was reduced in peripheral blood	(148)
Immunomodulatory agent	Thalidomide	TNF-α production and antigen-presenting ability of epidermal LCs were inhibited	(149)
	G-CSF	CD123+ pDCs were increased in lamina propria and gut mucosa	(150)
Hormones	Glucocorticoids	MHC-II expression on DCs was reduced	(151)
	Vitamin 1,25(OH)2 D	Pro-inflammatory DC activity was decreased	(152)
	Vitamin D3	Beneficial effects on monocytic precursors of mo-DCs *in vivo*	(153)
Thiopurine-based treatments	Azathioprine or 6-mercaptopurine	Migratory defects in autophagy-deficient DCs were restored	(154)
Biodrugs	Mesenchymal stem cells	Serum levels of pro-inflammatory cytokines (IL-1β, IL-6) were reduced Expression of CD80 and CD86 was decreased on mDCs IL-10 production was improved in pDCs	(155)
Probiotics	*Lactobacillus salivarius*, *Bacillus coagulans*, *Bacillus subtilis* and *Bifidobacterium bifidum (Bb)*	Expression of CD80, CD86 and integrin ß8 was enhanced TLR-4, TLR-9 and IL-12p40 expression was reduced TGF-β and IL-10 production was improved	(14)
	*Saccharomyces boulardii*	Th1 polarization induced by CD1c+CD11c+CD123-mDCs was inhibited TNF-α and IL- 6 production was reduced The expression of CD40, CD80, and CCR7 was reduced on mDCs	(156, 157)
	*Lactobacillus casei Shirota*	Gut DC ability to imprint homing molecules on T cells and IL-22 production were improved	(158)
	*Lactobacillus plantarum*	The function of altered gut DCs was reversed	(159)
Apheresis	Adacolumn apheresis	CD16 mDCs, pDCs, and TNF-a were reduced IL-10 production was increased	(160)
	Lymphocytapheresis	CD83+ DCs, IL-6, and IL-8 were downregulated	(161)
Saccharides	Lipopolysaccharides	Pro-inflammatory cytokine production and antigen-presenting ability for MHC-II were diminished when cultured with GLM *in vitro*	(162)
	Fructo-oligosaccharides	IL-10+, TLR2+, and TLR4+ DCs were increased IL-6+ DCs were reduced	(163, 164)
Nutraceuticals	Sulforaphane	Preventive/therapeutic applications due to its activating effect of the AMPK signaling pathway	(106)

### 6.1 Antibodies

The first successful therapies for IBD consisted of targeting TNF-α, including infliximab or adalimumab (148). Antibodies against IL-12/IL-23 p40 (risankizumab) and IL-23 p19 (ustekinumab) have been tested to reduce the effects in IBD (133). Other antibodies target the α4 chain of integrin heterodimers on leukocytes (e.g., natalizumab), α4β7 integrin, which may reduce the inflammation by blocking the recruitment of pro-inflammatory monocytes and DCs to the intestine (e.g., vedolizumab) (148) or immunomodulatory agents such as thalidomide (149) and G-CSF (150).

### 6.2 Glucocorticoids and thiopurine-based therapies

Alternative therapies such as the use of glucocorticoids can inhibit cytokine secretion, as well as both T cell and DC activation by reducing the expression of MHC-II molecules in UC (151). Others such as thiopurine-based treatments have demonstrated to restore the migratory defects in autophagy-deficient DCs, thus improving DC-T cell interactions and the cytoskeletal regulation (154). Moreover, mesenchymal stem cell (MSC) administration reduced serum levels of inflammatory cytokines (e.g., IL-1β, IL-6, and IL-12) in mice with DSS-induced UC, thus improving the phenotype and function of colon infiltrating DCs (155). In CD, MSCs not only decreased the expression of CD80 and CD86 on mDCs and the production of IL-12 and TNF-α, but also improved the production of IL-10 via (165).

### 6.3 Antibiotics and probiotics

Antibiotics reduce the concentration of gut lumen bacteria such as *Escherichia coli* strains, Bacteroides spp, and *Mycobacterium avium*, that have been linked, together with DCs, to chronic inflammation in IBD (166). Specifically, betalactam antibiotics have demonstrated to alter DC maturation in allergic patients via MAPK and NF-kB signaling pathways (167). IBD is also promoted by DC migration and maturation, so the targeting of DCs with betalactam antibiotics may improve clinical outcomes in the disease (115, 116, 168). Probiotics have also been successfully proposed to modulate the gut microbiota in IBD (169). In this sense, Ghavami et al. (2020) studied the role of *Lactobacillus salivarius*, *Bacillus coagulans*, *Bacillus subtilis* and *Bifidobacterium bifidum (Bb)*, concluding that the expression of CD80 and CD86 was enhanced by most of the probiotics in UC patients and only by *Bb* in CD patients. Also, DCs from UC patients increased the production of IL-10 and TGF-β and reduced the expression of TLR by using all probiotics except *Bb*, and DCs from CD patients increased the expression of integrin ß8 and reduced the expression of TLR-4, TLR-9, and IL-12p40 (14). *Saccharomyces boulardii* promoted epithelial restitution in IBD patients by improving IL-8 levels, inhibiting Th1 polarization induced by CD1c^+^CD11c^+^CD123^-^ mDCs, and reducing TNF-α and IL-6 levels, as well as the expression of CD40, CD80, and CCR7 on mDCs (156, 157). *Lactobacillus casei Shirota* restored the stimulatory role of DCs in UC patients (170, 171) by improving gut DC ability to imprint homing molecules on T cells and promoting IL-22 production (158). Furthermore, *Lactobacillus plantarum* has reversed the function of altered gut DCs in UC patients (159).

### 6.4 Apheresis

Selective granulocyte/monocyte apheresis (SGMA) has been tested to remove DCs from IBD patients (172). Adacolumn apheresis (AA) could lead to a higher tolerogenic status since a significantly lower level of lymphocytes, pDCs and mDCs has been found in acute UC (160). In addition, AA increased IL-10 and reduced circulating TNF-α and CD16 expression on both mDC and pDCs in UC patients (173). Lymphocytapheresis has demonstrated to be clinically safe in those patients and contributed to downregulate CD83^+^ DCs, IL-6, and IL-8 (161).

### 6.5 Vitamin D

Vitamin D also seems to have a role in the modulation of DCs. In fact, vitamin D metabolites are frequently used in protocols to develop therapeutic DC therapies for autoimmune diseases, such as IBD (174). Vitamin 1,25(OH)2 D has improved IBD outcomes, at least in part, by decreasing DC activity, inducing antimicrobial peptide secretion, and increasing the anti-/pro-inflammatory cytokine ratio (152). Vitamin D3 was positively associated with low disease activity in CD patients and had beneficial effects *in vivo* on the monocytic precursors of moDC (153).

### 6.6 Saccharides

In the same line, vitamin D deficiency has been suggested to contribute to the inflammatory process in CD based on data from *in vitro* experiments by stimulating mo-DC with lipopolysaccharides (LPS) (162). Conversely, LPS-activated DCs has been cultured with GLM, a luteolin derivative, downregulating pro-inflammatory cytokine production or antigen-presenting ability for MHC-II complex on DCs from UC (175). Other molecules with natural origin as fructo-oligosaccharides, have significantly increased the number of IL-10^+^, TLR2^+^, and TLR4^+^ DCs, and reduced IL-6^+^ DCs in CD patients, but the clinical benefit remains contradictory (163, 164).

### 6.7 Sulforaphane

Another molecule, sulforaphane, with anticancer properties (106), has preventive or therapeutic applications in some intestinal inflammatory diseases due to its activating effect of AMPK signaling pathway in mice (176), although more evidence would be necessary to confirm the role of this natural compound on DCs.

## 7 Concluding remarks

DCs have a crucial role in the establishing and maintaining immune homeostasis of the organism, because link innate and adaptive immunity since they initiate immune responses by taking up both antigens and pathogens, and migrating to secondary lymphoid organs, where DCs finally present molecules to naïve T cells, which are activated. This essential immune process is employed as therapeutic intervention tool to the cure or mitigate of many diseases such as cancer seeking to enhance the cytotoxic machinery of T cells to kill tumor cells. Conversely, under certain conditions, DCs have shown to play a key role in the induction and maintenance of chronic inflammation in other pathologies, including IBD, resulting in the so-called “yin-yang” role of DCs.

It is clear that not only DCs promote a pro-inflammatory state in the intestine, but also other recruited immune cells such as neutrophils, monocytes or macrophages. In this sense, monocytes and macrophages could be difficult to distinguish from some DC subsets because they express the same markers depending on their differentiation stage in the myeloid lineage. Even so, activated DCs have found to be accumulated at sites of intestinal inflammation expressing a wide variety of characteristics markers, including (but not limited to) CD80, CD86, CD103, CD83, IRF4 or TLRs, and producing cytokines such as IL-6, IL-8, IL-12, IL-23, TNF-α, which produce disruptions in the immune system and drive IBD progression.

Based on the existing evidence on the role of DCs in IBD, we strongly believe that this cell population can be considered as a good biomarker for the disease. In fact, most of *in vivo* and *in vitro* experiments have shown that DCs could be a valuable therapeutic target, since its depletion as well as the production of some cytokines (e.g., IL-10 or TGF-β) have been positively associated with good results, which could support the manipulation of DCs to generate DC-specific therapies. For that purpose, we would also need to fully understand the mechanisms that are promoted by DCs in the balance between immune cells, since the pro-inflammatory state in the intestine could be increased. In addition, further research is needed to better clarify the importance of some DCs markers in the disease (e.g., CD1c, CD11c, or CD123) since both their expression and lack on the cell surface have been associated with positive results in the disease. Moreover, the role of moDCs or infDCs and LCs has been less studied and may play a critical role in the pathogenesis of IBD, so those DCs subset would also need to be more investigated to reach innovative strategies to enhance their clinical efficacy in both IBD and cancer.

## Author contributions

CJ-C: Writing – original draft, Writing – review & editing. FP: Writing – review & editing. GA: Writing – review & editing. CS-M: Writing – review & editing. LC-M: Writing – review & editing. VS-M: Conceptualization, Supervision, Writing – review & editing. SL-E: Conceptualization, Supervision, Writing – review & editing.
